# Design and rationale of a routine clinical care pathway and prospective cohort study in older patients needing intensive treatment

**DOI:** 10.1186/s12877-020-01975-0

**Published:** 2021-01-07

**Authors:** Yara van Holstein, Floor J. van Deudekom, Stella Trompet, Iris Postmus, Anna Uit den Boogaard, Marjan J. T. van der Elst, Nienke A. de Glas, Diana van Heemst, Geert Labots, Mariëtte Altena, Marije Slingerland, Gerrit Jan Liefers, Frederiek van den Bos, Jessica M. van der Bol, Gerard J. Blauw, Johanneke E. A. Portielje, Simon P. Mooijaart

**Affiliations:** 1 Department of Internal Medicine, Section of Gerontology and Geriatrics, Leiden University Medical Center, PO box 9600, 2300 RC Leiden, The Netherlands; 2 Department of Medical Oncology, Leiden University Medical Center, Leiden, The Netherlands; 3 Department of Internal Medicine, Haga Hospital, The Hague, The Netherlands; 4 Department of Internal Medicine, Haaglanden Medical Center, The Hague, The Netherlands; 5 Department of Surgery, Leiden University Medical Center, Leiden, The Netherlands; 6 Department of Geriatrics, Reinier de Graaf Hospital, Delft, The Netherlands; 7 Institute for Evidence-based Medicine in Old Age (IEMO), Leiden, The Netherlands

**Keywords:** Older patients, Comprehensive geriatric assessment, Frailty, Geriatric oncology, Aortic valve replacement, Oesophageal cancer, Head and neck cancer, Colon cancer, Study protocol

## Abstract

**Background:**

Treatment decisions concerning older patients can be very challenging and individualised treatment plans are often required in this very heterogeneous group. In 2015 we have implemented a routine clinical care pathway for older patients in need of intensive treatment, including a comprehensive geriatric assessment (CGA) that was used to support clinical decision making. An ongoing prospective cohort study, the Triaging Elderly Needing Treatment (TENT) study, has also been initiated in 2016 for participants in this clinical care pathway, to study associations between geriatric characteristics and outcomes of treatment that are relevant to older patients. The aim of this paper is to describe the implementation and rationale of the routine clinical care pathway and design of the TENT study.

**Methods:**

A routine clinical care pathway has been designed and implemented in multiple hospitals in the Netherlands. Patients aged ≥70 years who are candidates for intensive treatments, such as chemotherapy, (chemo-)radiation therapy or major surgery, undergo frailty screening based on the Geriatric 8 (G-8) questionnaire and the Six-Item Cognitive Impairment Test (6CIT). If screening reveals potential frailty, a CGA is performed. All patients are invited to participate in the TENT study. Clinical data and blood samples for biomarker studies are collected at baseline. During follow-up, information about treatment complications, hospitalisations, functional decline, quality of life and mortality is collected. The primary outcome is the composite endpoint of functional decline or mortality at 1 year.

**Discussion:**

Implementation of a routine clinical care pathway for older patients in need of intensive treatment provides the opportunity to study associations between determinants of frailty and outcomes of treatment. Results of the TENT study will support individualised treatment for future patients.

**Trial registration:**

The study is retrospectively registered at the Netherlands Trial Register (NTR), trial number NL8107. Date of registration: 22-10-2019.

## Background

Clinical decision making in relation to older patients with an indication for intensive treatment can be very challenging. The rate of ageing differs between individuals, resulting in heterogeneity in physiological and functional characteristics, life expectancy and treatment tolerance [1, 2]. Older patients are underrepresented in clinical trials [3, 4] and due to strict inclusion criteria and the selective inclusion practiced by physicians, the majority of older trial participants is generally in relatively good health and has a good performance status [5, 6]. As a result, treatment decisions in older patients with poorer health status are not supported by scientific evidence. In addition, most endpoints of clinical trials are related to mortality and treatment toxicity [7], whereas older patients might prioritize functional and cognitive outcomes and quality of life over prolonged survival [8, 9]. Therefore, studying alternative adverse events such as early treatment discontinuation or unplanned hospitalisation may help to better weigh treatment risks and benefits and support the treatment decision process. Currently, however, detailed information on the determinants of relevant outcomes is often lacking.

A comprehensive geriatric assessment, performed by a geriatrician, is a multidisciplinary evaluation to assess the multiple problems of older patients and develop an integrated care plan for treatment and follow-up [10]. With an assessment of the four geriatric domains (somatic status, psychological status, functional status and social status) an overall view of the patients’ level of frailty is provided. Geriatric screening tests can be used to identify patients who might benefit from CGA [11]. A shorter geriatric assessment that focuses on identifying health issues can alternatively be performed by other physicians or nurses [12]. Although geriatric assessment is currently not part of routine clinical practice, it is known to predict treatment-related outcomes including survival, treatment toxicity [13, 14] and postoperative complications [15], and can support treatment choices and intensity [13, 16–18]. Several biomarkers may characterize the biological age of an older patient and predict outcomes, but have not yet been studied in a clinical context.

We have designed and implemented a routine clinical care pathway, which integrates geriatric assessment, to improve clinical decision making for older patients. The first aim of this paper is to describe the implementation and rationale of this pathway initiated in 2015. The second aim is to describe the rationale and design of the Triaging Elderly Needing Treatment (TENT) study, an ongoing prospective cohort study of participants in the clinical pathway.

## Methods

### Routine clinical care pathway

Starting in 2015, we have designed and implemented a routine clinical care pathway at Leiden University Medical Center (LUMC, Leiden), Haga Hospital (the Hague), Haaglanden Medical Center (HMC, the Hague), and the Reinier de Graaf Hospital (RdG, Delft). In this clinical pathway patients aged ≥70 years who are candidates for intensive treatment (e.g. surgery, chemotherapy, (chemo-)radiation therapy, immunotherapy or other cancer therapies) and are potentially frail are identified by geriatric screening and then undergo standardized CGA in the outpatient clinics at participating hospitals. CGA results are explained to the patient and discussed during a multidisciplinary team meeting to support individualised treatment decisions. Below we describe the elements of the care pathway and explain the rationale of the tests we chose. Table 1 provides a detailed description of the different tests, score ranges and cut-off scores used.

**Fig. 1:**
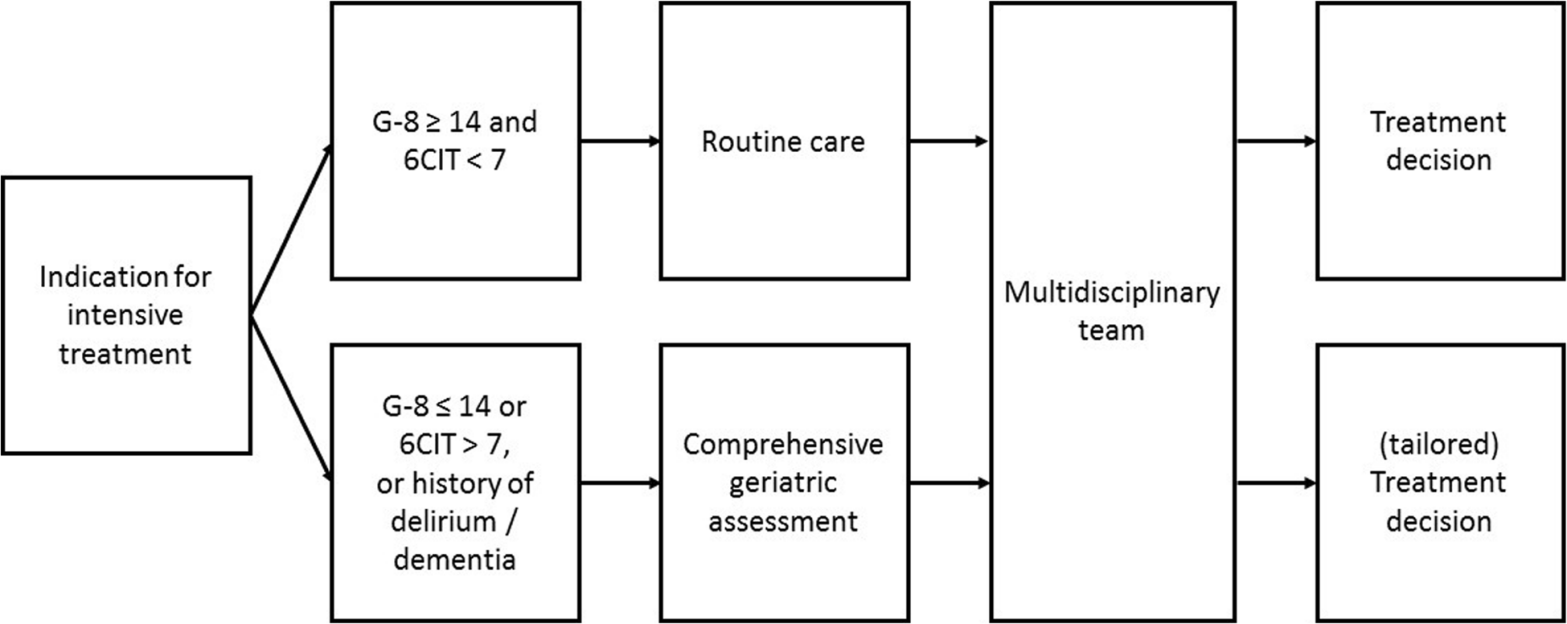
Overview of the routine clinical care pathway

- Patients
  Patients aged ≥70 years who are candidate for intensive treatment in cardiovascular, thoracic, orthopaedic and oncology outpatient clinics undergo geriatric screening.
- Geriatric screening
  A trained nurse uses geriatric screening to identify patients with potential frailty who may be in need of further evaluation by comprehensive geriatric assessment [11]. Geriatric screening consists of the Geriatric 8 (G-8) screening questionnaire [19] and the Six-Item Cognitive Impairment Test (6CIT) [20] and takes about 5 minutes to complete. The G-8 is an eight-item questionnaire developed for older cancer patients but also used in other populations. It covers multiple domains and places significant weight on nutritional status (47% of the total score). In a review by Decoster et al. the reported sensitivity to detect a need for further evaluation by geriatric assessment was over 80% in six studies [11]. The G-8 does not actually test cognition. Cognitive impairment is generally associated with adverse health outcomes such as delirium [37], a prolonged length of hospital stay [38] and subsequent mortality [38]. The 6CIT is a brief and simple cognitive test and correlates well with the Mini-Mental State Examination (MMSE) [21]. The original cut-off score of the 6CIT is ≥11 (i.e. MMSE 24), an alternative cut-off score is > 7 [20, 21]. When using the cut-off score of > 7, the reported sensitivity is 78.6% and the specificity is 100% [21]. We chose to use the alternative cut-off score > 7 to enhance sensitivity and identify more patients with potential cognitive impairment. Patients are referred for CGA when their G-8 score is ≤14 and/or the 6CIT is > 7, or if the patient has a history of delirium or dementia (Fig. 1).
- Comprehensive geriatric assessment
  In those patients with an abnormal geriatric screening a CGA is subsequently performed in the geriatric outpatient clinic. Time scheduled for this consultation is 60–90 min. Content:
  - ○ Somatic status
    The somatic status includes information about the current diagnosis and symptoms, medical history and medication use. Weight, height, body mass index (BMI), blood pressure, heart rate and orthostatic hypotension are measured and a complete physical examination is performed when indicated. Malnutrition is associated with mortality and functional dependency in different patient populations [39]. Nutritional status is assessed using the Mini Nutritional Assessment Short Form (MNA-SF®) [23]. The MNA-SF® is the test preferred by the Inspectorate of Public Health in the Netherlands [40].
  - ○ Psychological status
    Depressive symptoms are associated with outcomes such as mortality [41, 42] and functional decline [43]. The two-item Patient Health Questionnaire (PHQ-2) [24] is a short instrument used to screen for depression. This screening instrument is suitable since a score ≥ 3 shows a sensitivity of 83% and specificity of 92% for detecting major depression [24]. When further evaluation is needed, the Geriatric Depression Scale-15 (GDS-15) [25] is administered.
    Previous studies have shown an association between a higher level of optimism and a lower risk of cardiovascular events and all-cause mortality [44]. To measure optimism a three-item questionnaire is used that contains the three positively worded questions of the Life Orientation Test-Revised (LOT-R) [26].
    In the event of an abnormal 6CIT on geriatric screening, the Visual Association Test (VAT) [27] and the Clock Drawing Test [28] are performed to assess cognition. The VAT tests visual associative memory and the Clock Drawing Test assesses visuospatial and executive functioning. When indicated, a neurocognitive assessment including a full neuropsychological battery of tests is performed.
  - ○ Functional status
    Functional dependency is associated with mortality in both the general population and in hospitalised patients [45]. To explore patients’ functional status, the Activities of Daily Living score (Katz ADL) [29] and Instrumental Activities of Daily Living (Lawton IADL) [30] are assessed. The 6-item Katz ADL was chosen because it is already administered to all hospitalised patients aged ≥70 years as part of a mandatory national Dutch Safety Management System (Veiligheid Management Systeem Kwetsbare ouderen, VMS) and is suitable for extended follow-up. Furthermore, we previously successfully used the Katz ADL to follow over 2600 older patients who visited the Emergency Department [46, 47]. The 8-item Lawton IADL focuses on more complex activities of daily living. The aim of these questionnaires is to assess the overall (representative) functional status and is not based on (sub)acute functional decline prior to clinical evaluation. Furthermore, a 4-m gait speed measurement and handgrip strength are performed to assess physical capacity. Slow gait speed [31, 48] and poor handgrip strength [49, 50] are associated with outcomes such as mortality, disability and cognitive decline.
  - ○ Social status
    The patients’ social status is explored by asking about living arrangements (independent, institutionalized, hospitalised), the availability of a formal caregiver and level of support (number of days with home care).
  - ○ Quality of life
    Health-related quality of life is associated with mortality and functional decline in older hospitalised patients [51] and is measured using the EuroQol five dimensions questionnaire (EQ-5D-3L), including the visual analogue scale (EQ-VAS) [35]. Since the EQ-VAS is part of the outcome measures of the TENT study and is collected by telephone at follow-up, we chose to use a verbal description of the EQ-VAS and register the patients’ verbal answer on a numerical rating scale. Previous studies have shown comparable results between telephone administration of the EQ-5D and EQ-VAS and face-to-face administration [52] and patient-completed forms [53].
- Clinical decision making
  During a multidisciplinary team meeting different treatment options are considered, including standard care, less intensive treatment options and best supportive care. Information obtained from the comprehensive geriatric assessment, the remaining life expectancy, expected effect of different forms of treatment on relevant outcomes for older patients and patient preferences are taken into account. Patient preferences are assessed by asking the patients’ perspective on possible treatment goals, e.g. prolonged survival, maintaining independence, reducing symptoms or other personal goals. Less intensive treatment is proposed when the CGA indicates frailty and hence an increased risk of functional decline. Treatment recommendations that are formulated during multidisciplinary team meetings are again discussed with the patient during the final treatment decision consult, emphasizing patient perspective and the predictive value of existing geriatric impairments on relevant outcomes. This entire process results in individualised treatment decisions.

**Table 1:** Description of different tests

Test	Explanation	Scores
	**Geriatric screening**	
G-8 [19]	8-item screening test. Assesses domains of nutritional status, mobility, neuropsychological problems, medication use, self-rated health status and age	Score ranges from 0 to 17, lower score indicates more impairment, cut-off score ≤ 14
6CIT [20, 21]	6-item cognitive screening test. One memory, two attention and three orientation questions	Score ranges from 0 to 28, higher score indicates more significant cognitive impairment, cut-off score > 7
	**Comprehensive geriatric assessment**	
***Somatic status***		
Medical history	Polypharmacy, multi-morbidity using CCI [22]: 16 medical condition of which 3 are stratified according to severity	Score ranges from 0 to 33, higher score indicates more comorbidities
Physical measurement	Weight, height, BMI, blood pressure, heart rate, orthostatic hypotension, complete physical examination on indication	N/A
MNA-SF® [23]	6-item screening test. Assesses loss of appetite, weight loss, BMI, mobility, the occurrence of stress or an acute disease and neuropsychological problems	Score ranges from 0 to 14, lower score indicates greater risk of malnutrition, cut-off score ≤ 11
***Psychological status***		
PHQ-2 [24]	2-item screening test for depression	Score ranges from 0 to 6, higher score indicates more depressive symptoms, cut-off score ≥ 3
GDS-15 [25]	15-item questionnaire. Assesses depressive symptoms	Score ranges from 0 to 15, higher score indicates more depressive symptoms, cut-off score ≥ 5
Optimism questionnaire [26]	3-item questionnaire. Assesses optimism on a 5-point Likert scale: 0 corresponds to “strongly disagree” and 4 to “strongly agree”	Score ranges from 0 to 12, higher score indicates greater optimism
VAT [27]	Learning task that assesses visual associative memory	Score ranges from 0 to 12, lower score indicates more cognitive impairment
Clock drawing [28]	Cognitive test that assesses visuospatial and executive functioning	Score ranges from 0 to 14, lower score indicates more executive impairment, cut-off score < 10
***Functional status***		
Katz ADL [29]	6-item questionnaire. Assesses bathing, dressing, toileting, transfers, continence and feeding	Score ranges from 0 to 6, higher score indicates greater dependency
Lawton IADL [30]	8-item questionnaire. Assesses more complex independent living skills: ability to use a phone, shopping, food preparation, housekeeping, laundry, mode of transportation, responsibility for personal medications, ability to handle finances	Score ranges from 0 to 8, lower score indicates greater dependency
Gait speed [31, 32]	Timed 4-m walking test	Lower score represents slow gait speed, cut-off speed ≤0.8 m/s
Handgrip strength [33, 34]	Handgrip strength measurement, using a Jamar Handheld Dynamometer. Best of 3 measurements using the dominant hand	Reference values depend on age and gender
***Social status***	Living arrangement (independent, institutionalised, hospitalised), the availability of a caregiver, hours of (home)care	N/A
***Quality of life***		
EQ-5D-3L [35, 36]	5-item questionnaire. Assesses health-related quality of life exploring five dimensions: mobility, self-care, daily activities, pain/complaints, mood. Three possible levels of answers: no problems, some problems, extreme problems	An index score is calculated, score ranges from − 0.33 to 1.0. Score < 0 represents worse than dead and 1 represents full health
EQ-VAS [35, 36]	Verbal description of an overall health state visual analogue scale, registered on a numerical rating scale	Score ranges from 0 to 100, higher score indicates higher health-related quality of life

*Abbreviations*: *6CIT* 6 Item Cognitive Impairment Test, *ADL* Activities of Daily Living, *BMI* Body mass index, *CCI* Charlson Comorbidity Index, *EQ-5D-3L* EuroQol five dimensions three levels questionnaire, *EQ-VAS* EuroQol Visual Analogue Scale, *G-8* Geriatric eight, *GDS-15* Geriatric Depression Scale-15, *IADL* Instrumental Activities of Daily Living, *MNA-SF®* Mini Nutritional Assessment Short Form, *N/A* Not applicable, *PHQ-2* Patient Health Questionnaire, *VAT* Visual Association Test

### TENT study

The TENT study is embedded in the routine clinical care pathway, with the aim of developing prediction models to predict the outcome of various intensive treatments. The primary outcome is the composite endpoint of functional decline or mortality at 1 year. We hypothesize that elements of comprehensive geriatric assessment predict outcomes of intensive treatment in older patients and support treatment decisions.

We defined the following objectives:

1. To study the prevalence of geriatric impairments in older patients needing various intensive treatments.
2. To study the incidence of adverse health outcomes (mortality, functional decline, reduced quality of life) at 6 and 12 months.
3. To study associations between geriatric impairments and adverse health outcomes.
4. To study associations between biomarkers and determinants of frailty and adverse health outcomes.
5. To develop models to predict outcomes, containing geriatric determinants and biomarkers.

Below we describe the study design, participants, data collection and outcomes of interest and the statistical analyses that will be carried out.

- Study design
  The TENT study is a prospective cohort study that commenced on 1st February 2016 in the aforementioned hospitals. Inclusion is still ongoing. Patients in the care pathway are asked to participate in the present study, in which we collect clinical data and additional blood samples at baseline. Participants are followed for 1 year.
- Participants
  In order to assemble a representative cohort it is important that all patients in the clinical care pathway actually participate, including those patients without signs of frailty during geriatric screening. Consequently, all patients are invited to participate in the TENT study and are subsequently assessed for eligibility based on the criteria aged ≥70 years and candidate for intensive treatment, including surgery, chemotherapy, (chemo-)radiation therapy, immunotherapy or other cancer therapies. Participants who are not able to understand the Dutch language, or are not able to provide informed consent and have no proxy available, are excluded.
  When geriatric screening indicates potential frailty, the patient is referred to the geriatric outpatient clinic for comprehensive geriatric assessment and invited to participate. Patients without signs of frailty during geriatric screening are contacted by telephone for inclusion. The Medical Ethics Committee of the LUMC issued a ‘certificate of no objection’ for retrospective data collection of patients with the same diagnosis not included in the TENT study. This means we will be able to determine whether included patients are representative of the overall patient population in terms of baseline characteristics, treatment administered, and selected outcomes (mortality, treatment complications).
- Data collection
  - ○ Baseline
    The following data are collected from the digital patient files: medical history, medication use, smoking and alcohol status and history, level of education, multi-morbidity using the Charlson Comorbidity Index (CCI) [22], diagnosis that indicated intensive treatment, treatment choice, laboratory tests, geriatric screening, comprehensive geriatric assessment and in case of a malignancy, WHO performance status, tumour characteristics and stage. When a participant is not referred to the geriatric outpatient clinic, a short geriatric assessment is administered by telephone by a research nurse or researcher. This geriatric assessment includes psychological status (PHQ-2, optimism questionnaire), functional status (Katz ADL, Lawton IADL), social status and quality of life (EQ-5D-3L and EQ-VAS). Physical capacity tests are not performed.
  - ○ Follow-up
    Participants are contacted by telephone for follow-up at 6 and 12 months after the start of treatment. The following data are collected: Katz ADL, Lawton IADL, EQ-5D-3L and EQ-VAS, and social status. In case a participant is not able to answer the questions, a proxy is allowed to answer all questions except the EQ-VAS. The proxy is registered as contact in the digital patient file, or another caregiver involved in daily care is asked.
  - ○ Biomaterial
    At baseline blood samples are collected to study biomarkers of ageing. These samples consist of two gel tubes (8.5 cc), one tube of EDTA plasma (10 cc), and one sodium citrate tube (4.5 ml). We plan to use several methods to measure biological ageing, including algorithms that are based on routinely collected blood chemistry data, measurement of metabolomics, and epigenetics [54].
  - ○ Data management
    Data are recorded on Case Record Forms, encrypted and stored in an electronic data management system (Castor EDC [55]), in accordance to General Data Protection Regulations (GDPR).
- Outcomes
  The primary outcome is the composite endpoint of functional decline or mortality at 1 year. Data on the following endpoints are currently being collected:
  - ○ All-cause mortality, by consulting municipal registries (in Dutch: Basisregistratie Personen).
  - ○ Functional status at 6 and 12 months after treatment initiation. Functional improvement is defined as an at least one-point decrease in Katz ADL compared to baseline. Functional decline is defined as at least one-point increase in Katz ADL compared to baseline or a new institutionalization.
  - ○ Change in quality of life between baseline and 6 and 12 months follow-up based on the EQ-5D-3L index score and EQ-VAS.
  - ○ Complications during hospital admission or treatment, such as infections, delirium, re-operation, grade 3–5 toxicity of chemotherapy, radiation therapy or other cancer therapy, early treatment discontinuation, or adjustment of treatment intensity. This information is obtained from digital patient files.
  - ○ Total length of hospital stay, defined as the number of days between hospital admission for intensive treatment (surgery) and discharge. This information is obtained from digital patient files.
  - ○ Unplanned admission to an intensive care unit. This information is obtained from digital patient files.
  - ○ Unplanned hospital admission. This information is obtained from digital patient files.
- Statistical analysis
  Statistical analysis will be performed using SPSS software version 25 or STATA version 14. Determinants and endpoints will be tabulated to gain insight into data and regression models (Cox regression, linear regression and linear mixed models, binary logistic regression) used to study associations between determinants of endpoints, taking into account potential confounding. Excessive testing can be avoided by formulating hypotheses before the data analysis, reducing false-positive findings. When necessary, correction for multiple testing will be applied. Moreover, to illustrate the clinical significance of associations, results will be compared to the minimal clinically important difference. Table 2 shows the minimal clinically important differences (MCID) of the Katz ADL [56], Lawton IADL [56], EQ-5D-3L [57] and EQ-VAS [57] as reported in previous studies that most closely resemble our study population.

**Table 2:** Minimal clinically important difference (MCID) of follow-up tests

Test	Scale	Minimal important difference
Katz ADL	0–6	0.18–0.47 [56]
Lawton IADL	0–8	0.31–0.77 [56]
EQ-5D-3L	−0.33 – 1.0	0.06–0.08 [57]
EQ-VAS	0–100	7 [57]

*Abbreviations ADL* Activities of Daily Living, *EQ-5D-3L* EuroQol five dimensions three levels questionnaire, *EQ-VAS* EuroQol Visual Analogue Scale, *IADL* Instrumental Activities of Daily Living
  We will also transform predictive values from the multivariate models into individual risk scores, predicting the selected endpoint using Receiver Operating Curves (ROC) and their area under the curve (AUC, also called c-statistic). Sensitivity, specificity, positive and negative predictive power will also be assessed. Several techniques are available to evaluate models. We intend to use bootstrapping methods for internal validation.
- Sample size calculation
  The number of participants differs per disease, but we aim to have sufficient power to predict adverse health outcomes in the various groups. We will carry out a formal power calculation per research question depending on the disease, determinant and outcome studied. An example of a sample size calculation is provided for a prediction model with the composite outcome of functional decline or 1-year mortality. Functional decline is defined as an at least one point increase in Katz ADL score or new institutionalization at 1-year follow-up. To reduce the risk of false positive findings (predictors) the so-called ‘EPV (events per variable) 1 to 10 rule of thumb’ is often applied. This rule suggests that at least 10 events per candidate predictor are needed for reliable prediction modelling [58, 59]. As we expect the model to include 8 predictors, at least 80 patients with the event of interest will be needed. When the incidence of the composite outcome in a certain patient population is 30% and the drop-out rate is 10%, the target sample size for the prediction model would be 294 patients.
- Ethics approval and consent to participate
  The TENT study protocol was approved by the Medical Ethics Committee (METC) at Leiden University Medical Center. All participants or a proxy provided written informed consent.

## Discussion

This paper describes the implementation and rationale of a routine clinical care pathway and design of the TENT study.

**Fig. 2:**
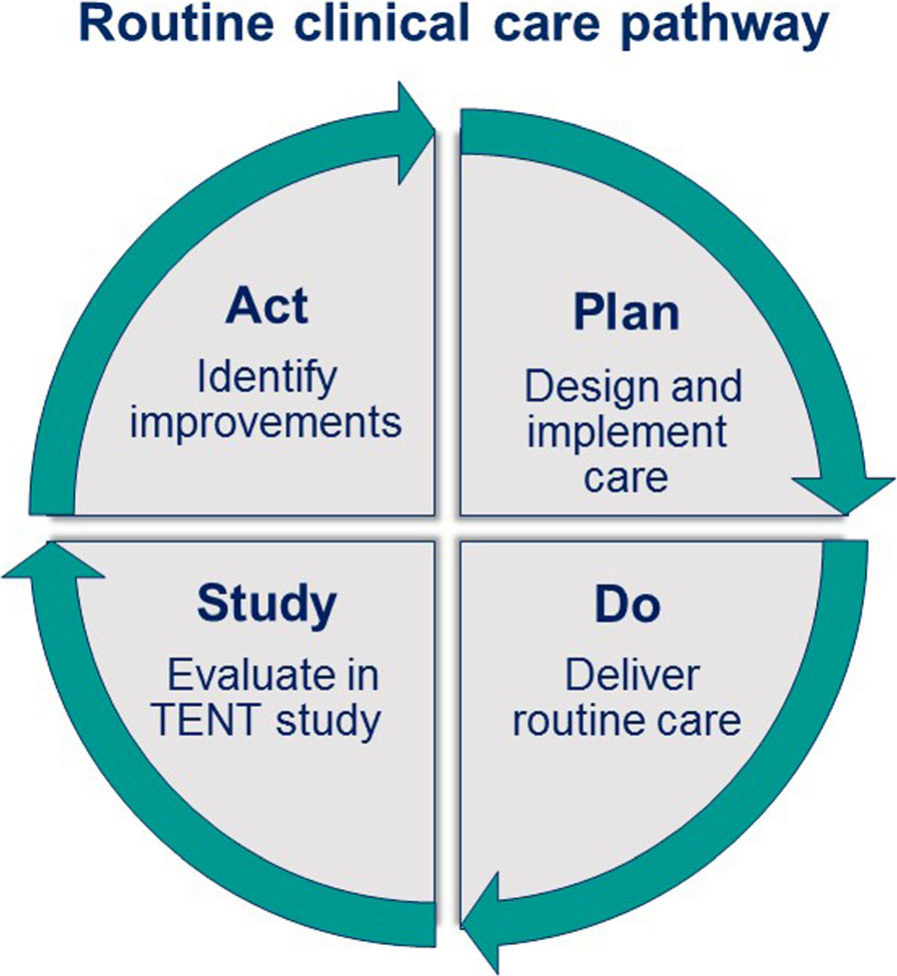
Interplay between routine clinical care pathway and TENT study in a Plan-Do-Study-Act (PDSA) cycle

Implementation of a routine clinical care pathway provides the opportunity to prospectively study associations between determinants of frailty and outcomes of treatment. These results are continuously evaluated to improve care. Figure 2 illustrates the interplay between the care pathway and TENT study using a Plan-Do-Study-Act (PDSA) cycle. As one example of this interplay, van Deudekom et al. showed that nutritional status and mobility were determinants of 1-year mortality in older patients with head and neck cancer at the LUMC [60]. These results are now integrated into treatment advice in current daily practice.

Due to heterogeneity in the older patient population, design of an individualised treatment plan will require better approaches to patient characterization. Despite good evidence supporting its important role in formulating treatment decisions and improving communication regarding age related concerns [61], CGA is not yet part of routine clinical care and in the majority of clinical trials it is either not performed or not reported [62]. In addition, biomarkers (of ageing) might also help to individualise treatment. An example of incorporating routinely measured biomarkers into a prediction tool is the Cancer and Aging Research Group’s (CARG) Chemo-Toxicity Calculator [14], which combines serum creatinine and haemoglobin, clinical data and geriatric parameters to predict risk of chemotherapy toxicity. Recent studies have shown promising results in measuring biological age and predicting adverse health outcomes in population-based cohorts [63–65]. However, their added value to a comprehensive geriatric assessment in clinical practice is still uncertain.

Randomised clinical trials are considered the highest level of evidence. However, only a small proportion of current randomised clinical trials focus on older patients [66, 67]. As one example of a randomised clinical trial, Hall et al. showed that a lower dose of chemotherapy in frail patients with advanced gastroesophageal cancer is non-inferior in terms of progression-free survival and resulted in less toxicity [68]. In the absence of sufficient clinical trials, observational studies can contribute to fill the evidence gap in older patients [69]. Prospective cohort studies that include geriatric assessment at baseline together with relevant outcomes will provide valuable real-life based data, an example of which is the ‘Carolina Senior’ registry (NCT01137825). The TENT study will also contribute to filling the evidence gap by including representative older patients, phenotyping their psychological, social and physical status and studying relevant endpoints that have rarely been addressed in previous studies. This will help to determine which patients may benefit from intensive treatment and reveal the impact of intensive treatment on alternative study endpoints, such as functional decline, quality of life and early treatment discontinuation. The TENT study will also contribute to knowledge on the pathophysiological mechanisms that drive ageing and disease by studying the association between geriatric parameters and biomarkers of ageing. We aim to combine geriatric assessment variables and biomarkers to develop models to predict treatment outcomes that are feasible to implement in clinical practice, and thereby support treatment decisions for future patients. Ultimately, the scientific evidence showing that this approach leads to improvement in relevant study endpoints should be derived from a randomised clinical trial, for example a step-wedge design.

Finally, collaboration of multiple hospitals in the study ensures a uniform approach to older patients, based on use of the same geriatric screening tests and instruments for geriatric assessment. This will improve communication between clinicians in different hospitals and with general practitioners. Moreover, it promotes inclusion of sufficient numbers of older patients in observational studies and provides opportunities for data sharing and the validation of tests and prediction tools [70].

## Conclusion

Implementation of a routine clinical care pathway for older patients in need of intensive treatments provides the opportunity to study associations between determinants of frailty and outcomes of treatment. Results of the TENT study will support individualised treatment for future patients.

## Data Availability

Not applicable.

## Abbreviations

6CIT: Six Item Cognitive Impairment Test
ADL: Activities of Daily Living
AUC: Area under the curve
BMI: Body mass index
CARG: Cancer and Aging Research Group
CCI: Charlson Comorbidity Index
EPV: Events per variable
EQ-5D: EuroQol five dimensions questionnaire
EQ-5D-3L: EuroQol five dimensions three levels questionnaire
EQ-VAS: EuroQol Visual Analogue Scale
G-8: Geriatric eight
GDPR: General Data Protection Regulation
GDS-15: Geriatric Depression Scale-15
HMC: Haaglanden Medical Center
IADL: Instrumental Activities of Daily Living
IEMO: Institute for Evidence-based Medicine in Old Age
LOT-R: Life Orientation Test-Revised
LUMC: Leiden University Medical Center
MCID: Minimal Clinically Important Difference
METC: Medical Ethics Committee
MMSE: Mini-Mental State Examination
MNA-SF®: Mini Nutritional Assessment Short Form
NTR: Netherlands Trial Register
PDSA: Plan-Do-Study-Act
PHQ-2: Patient Health Questionnaire
ROC: Receiver Operating Curve
RdG: Reinier de Graaf hospital
TENT: Triage of Elderly Needing Treatment
VAT: Visual Association Test
VMS: Veiligheid Management Systeem (Dutch for Safety Management System)
VOILA: Vitality Oriented Innovations for the Lifecourse of the Ageing Society

## References

[1] Lowsky DJ, Olshansky SJ, Bhattacharya J, Goldman DP (2014). Heterogeneity in healthy aging. J Gerontol A Biol Sci Med Sci.

[2] Soto-Perez-de-Celis E, Li D, Yuan Y, Lau YM, Hurria A (2018). Functional versus chronological age: geriatric assessments to guide decision making in older patients with cancer. Lancet Oncol.

[3] Hurria A, Levit LA, Dale W, Mohile SG, Muss HB, Fehrenbacher L (2015). Improving the evidence base for treating older adults with cancer: American Society of Clinical Oncology statement. J Clin Oncol.

[4] Singh H, Kanapuru B, Smith C, Fashoyin-Aje LA, Myers A, Kim G (2017). FDA analysis of enrollment of older adults in clinical trials for cancer drug registration: a 10-year experience by the U.S. Food Drug Adm.

[5] Van de Water W, Bastiaannet E, Van de Velde CJ, Liefers GJ (2011). Inclusion and analysis of older adults in RCTs. J Gen Intern Med.

[6] van de Water W, Kiderlen M, Bastiaannet E, Siesling S, Westendorp RG, van de Velde CJ (2014). External validity of a trial comprised of elderly patients with hormone receptor-positive breast cancer. J Natl Cancer Inst.

[7] Wildiers H, Mauer M, Pallis A, Hurria A, Mohile SG, Luciani A (2013). End points and trial design in geriatric oncology research: a joint European organisation for research and treatment of cancer--alliance for clinical trials in oncology--international society of geriatric oncology position article. J Clin Oncol.

[8] Fried TR, Bradley EH, Towle VR, Allore H (2002). Understanding the treatment preferences of seriously ill patients. N Engl J Med.

[9] Rietjens JA, van der Heide A, Voogt E, Onwuteaka-Philipsen BD, van der Maas PJ, van der Wal G (2005). Striving for quality or length at the end-of-life: attitudes of the Dutch general public. Patient Educ Couns.

[10] Rubenstein LZ, Stuck AE, Siu AL, Wieland D (1991). Impacts of geriatric evaluation and management programs on defined outcomes: overview of the evidence. J Am Geriatr Soc.

[11] Decoster L, Van Puyvelde K, Mohile S, Wedding U, Basso U, Colloca G (2015). Screening tools for multidimensional health problems warranting a geriatric assessment in older cancer patients: an update on SIOG recommendations dagger. Ann Oncol.

[12] Hamaker ME, Wildes TM, Rostoft S (2017). Time to stop saying geriatric assessment is too time consuming. J Clin Oncol.

[13] Wildiers H, Heeren P, Puts M, Topinkova E, Janssen-Heijnen ML, Extermann M (2014). International Society of Geriatric Oncology consensus on geriatric assessment in older patients with cancer. J Clin Oncol.

[14] Hurria A, Togawa K, Mohile SG, Owusu C, Klepin HD, Gross CP (2011). Predicting chemotherapy toxicity in older adults with cancer: a prospective multicenter study. J Clin Oncol.

[15] Feng MA, McMillan DT, Crowell K, Muss H, Nielsen ME, Smith AB (2015). Geriatric assessment in surgical oncology: a systematic review. J Surg Res.

[16] Pilotto A, Cella A, Pilotto A, Daragjati J, Veronese N, Musacchio C (2017). Three decades of comprehensive geriatric assessment: evidence coming from different healthcare settings and specific clinical conditions. J Am Med Dir Assoc.

[17] Caillet P, Laurent M, Bastuji-Garin S, Liuu E, Culine S, Lagrange JL (2014). Optimal management of elderly cancer patients: usefulness of the comprehensive geriatric assessment. Clin Interv Aging.

[18] Partridge JS, Harari D, Martin FC, Peacock JL, Bell R, Mohammed A (2017). Randomized clinical trial of comprehensive geriatric assessment and optimization in vascular surgery. Br J Surg.

[19] Bellera CA, Rainfray M, Mathoulin-Pelissier S, Mertens C, Delva F, Fonck M (2012). Screening older cancer patients: first evaluation of the G-8 geriatric screening tool. Ann Oncol.

[20] Tuijl JP, Scholte EM, de Craen AJ, van der Mast RC (2012). Screening for cognitive impairment in older general hospital patients: comparison of the six-item cognitive impairment test with the mini-mental state examination. Int J Geriatr Psychiatry.

[21] Brooke P, Bullock R (1999). Validation of a 6 item cognitive impairment test with a view to primary care usage. Int J Geriatr Psychiatry.

[22] Charlson ME, Pompei P, Ales KL, MacKenzie CR (1987). A new method of classifying prognostic comorbidity in longitudinal studies: development and validation. J Chronic Dis.

[23] Guigoz Y, Lauque S, Vellas BJ (2002). Identifying the elderly at risk for malnutrition. The mini nutritional assessment. Clin Geriatr Med.

[24] Kroenke K, Spitzer RL, Williams JB (2003). The patient health questionnaire-2: validity of a two-item depression screener. Med Care.

[25] Yesavage JA, Sheikh JI (1986). 9/geriatric depression scale (GDS). Clin Gerontol.

[26] Scheier MF, Carver CS, Bridges MW (1994). Distinguishing optimism from neuroticism (and trait anxiety, self-mastery, and self-esteem): a reevaluation of the life orientation test. J Pers Soc Psychol.

[27] Lindeboom J, Schmand B, Tulner L, Walstra G, Jonker C (2002). Visual association test to detect early dementia of the Alzheimer type. J Neurol Neurosurg Psychiatry.

[28] Royall DR, Cordes JA, Polk M (1998). CLOX: an executive clock drawing task. J Neurol Neurosurg Psychiatry.

[29] Katz S, Ford AB, Moskowitz RW, Jackson BA, Jaffe MW (1963). Studies of illness in the aged. The index of Adl: a standardized measure of biological and psychosocial function. JAMA..

[30] Lawton MP, Brody EM (1969). Assessment of older people: self-maintaining and instrumental activities of daily living. Gerontologist..

[31] Pamoukdjian F, Paillaud E, Zelek L, Laurent M, Levy V, Landre T (2015). Measurement of gait speed in older adults to identify complications associated with frailty: a systematic review. J Geriatr Oncol.

[32] Bohannon RW, Andrews AW (2011). Normal walking speed: a descriptive meta-analysis. Physiotherapy..

[33] Bohannon RW, Peolsson A, Massy-Westropp N, Desrosiers J, Bear-Lehman J (2006). Reference values for adult grip strength measured with a Jamar dynamometer: a descriptive meta-analysis. Physiotherapy..

[34] Dodds RM, Syddall HE, Cooper R, Benzeval M, Deary IJ, Dennison EM (2014). Grip strength across the life course: normative data from twelve British studies. PLoS One.

[35] EuroQol G (1990). EuroQol--a new facility for the measurement of health-related quality of life. Health Policy.

[36] Lamers LM, McDonnell J, Stalmeier PF, Krabbe PF, Busschbach JJ (2006). The Dutch tariff: results and arguments for an effective design for national EQ-5D valuation studies. Health Econ.

[37] Robinson TN, Raeburn CD, Tran ZV, Angles EM, Brenner LA, Moss M (2009). Postoperative delirium in the elderly: risk factors and outcomes. Ann Surg.

[38] Reynish EL, Hapca SM, De Souza N, Cvoro V, Donnan PT, Guthrie B (2017). Epidemiology and outcomes of people with dementia, delirium, and unspecified cognitive impairment in the general hospital: prospective cohort study of 10,014 admissions. BMC Med.

[39] O'Shea E, Trawley S, Manning E, Barrett A, Browne V, Timmons S (2017). Malnutrition in hospitalised older adults: a multicentre observational study of prevalence, associations and outcomes. J Nutr Health Aging.

[40] Inspectie Gezondheidszorg en Jeugd MvV, Welzijn en Sport. Basisset Medisch Specialistische Zorg Kwaliteitsindicatoren 2019. file:///H:/Roaming/Downloads/IGJ+Basisset+MSZ+2019.pdf. Accessed 27 Jan 2020.

[41] Drudi LM, Ades M, Turkdogan S, Huynh C, Lauck S, Webb JG (2018). Association of Depression with mortality in older adults undergoing transcatheter or surgical aortic valve replacement. JAMA Cardiol.

[42] Covinsky KE, Kahana E, Chin MH, Palmer RM, Fortinsky RH, Landefeld CS (1999). Depressive symptoms and 3-year mortality in older hospitalized medical patients. Ann Intern Med.

[43] Dunham NC, Sager MA (1994). Functional status, symptoms of depression, and the outcomes of hospitalization in community-dwelling elderly patients. Arch Fam Med.

[44] Rozanski A, Bavishi C, Kubzansky LD, Cohen R (2019). Association of optimism with cardiovascular events and all-cause mortality: a systematic review and meta-analysis. JAMA Netw Open.

[45] Stineman MG, Xie D, Pan Q, Kurichi JE, Zhang Z, Saliba D (2012). All-cause 1-, 5-, and 10-year mortality in elderly people according to activities of daily living stage. J Am Geriatr Soc.

[46] de Gelder J, Lucke JA, Blomaard LC, Booijen AM, Fogteloo AJ, Anten S (2018). Optimization of the APOP screener to predict functional decline or mortality in older emergency department patients: cross-validation in four prospective cohorts. Exp Gerontol.

[47] Blomaard LC, Lucke JA, de Gelder J, Anten S, Alsma J, Schuit SCE (2020). The APOP screener and clinical outcomes in older hospitalised internal medicine patients. Neth J Med.

[48] Studenski S, Perera S, Patel K, Rosano C, Faulkner K, Inzitari M (2011). Gait speed and survival in older adults. JAMA..

[49] Taekema DG, Gussekloo J, Maier AB, Westendorp RG, de Craen AJ (2010). Handgrip strength as a predictor of functional, psychological and social health. A prospective population-based study among the oldest old. Age Ageing.

[50] Ling CH, Taekema D, de Craen AJ, Gussekloo J, Westendorp RG, Maier AB (2010). Handgrip strength and mortality in the oldest old population: the Leiden 85-plus study. CMAJ..

[51] Parlevliet JL, MacNeil-Vroomen J, Buurman BM, de Rooij SE, Bosmans JE (2016). Health-related quality of life at admission is associated with postdischarge mortality, functional decline, and institutionalization in acutely hospitalized older medical patients. J Am Geriatr Soc.

[52] McPhail S, Lane P, Russell T, Brauer SG, Urry S, Jasiewicz J (2009). Telephone reliability of the Frenchay activity index and EQ-5D amongst older adults. Health Qual Life Outcomes.

[53] Chatterji R, Naylor JM, Harris IA, Armstrong E, Davidson E, Ekmejian R (2017). An equivalence study: are patient-completed and telephone interview equivalent modes of administration for the EuroQol survey?. Health Qual Life Outcomes.

[54] Partridge L, Deelen J, Slagboom PE (2018). Facing up to the global challenges of ageing. Nature..

[55] Castor EDC (2019). Castor Electronic Data Capture 2019.

[56] Suijker JJ, van Rijn M, Ter Riet G, Moll van Charante EP, de Rooij SE, Buurman BM (2017). Minimal important change and minimal detectable change in activities of daily living in community-living older people. J Nutr Health Aging.

[57] Pickard AS, Neary MP, Cella D (2007). Estimation of minimally important differences in EQ-5D utility and VAS scores in cancer. Health Qual Life Outcomes.

[58] Peduzzi P, Concato J, Feinstein AR, Holford TR (1995). Importance of events per independent variable in proportional hazards regression analysis. II. Accuracy and precision of regression estimates. J Clin Epidemiol.

[59] Peduzzi P, Concato J, Kemper E, Holford TR, Feinstein AR (1996). A simulation study of the number of events per variable in logistic regression analysis. J Clin Epidemiol.

[60] van Deudekom FJ, van der Velden LA, Zijl WH, Schimberg AS, Langeveld AP, Slingerland M (2019). Geriatric assessment and 1-year mortality in older patients with cancer in the head and neck region: a cohort study. Head Neck.

[61] Mohile SG, Epstein RM, Hurria A, Heckler CE, Canin B, Culakova E (2019). Communication with older patients with cancer using geriatric assessment: a cluster-randomized clinical trial from the National Cancer Institute Community Oncology Research Program. JAMA Oncol.

[62] van Deudekom FJ, Postmus I, van der Ham DJ, Pothof AB, Broekhuizen K, Blauw GJ (2017). External validity of randomized controlled trials in older adults, a systematic review. PLoS One.

[63] Fischer K, Kettunen J, Wurtz P, Haller T, Havulinna AS, Kangas AJ (2014). Biomarker profiling by nuclear magnetic resonance spectroscopy for the prediction of all-cause mortality: an observational study of 17,345 persons. PLoS Med.

[64] Deelen J, Kettunen J, Fischer K, van der Spek A, Trompet S, Kastenmuller G (2019). A metabolic profile of all-cause mortality risk identified in an observational study of 44,168 individuals. Nat Commun.

[65] Liu Z, Kuo PL, Horvath S, Crimmins E, Ferrucci L, Levine M (2018). A new aging measure captures morbidity and mortality risk across diverse subpopulations from NHANES IV: a cohort study. PLoS Med.

[66] Abbasi J. Older patients (still) left out of cancer clinical trials. JAMA. 2019. 10.1001/jama.2019.17016. 10.1001/jama.2019.17016 31647507

[67] Broekhuizen K, Pothof A, de Craen AJ, Mooijaart SP (2015). Characteristics of randomized controlled trials designed for elderly: a systematic review. PLoS One.

[68] Hall P, Swinson D, Waters J, Wadsley J, Falk S, Roy R (2019). Optimizing chemotherapy for frail and elderly patients (pts) with advanced gastroesophageal cancer (aGOAC): The GO2 phase III trial. J Clin Oncol.

[69] Mooijaart SP, Broekhuizen K, Trompet S, de Craen AJ, Gussekloo J, Oleksik A (2015). Evidence-based medicine in older patients: how can we do better?. Neth J Med.

[70] Oud FMM, de Rooij S, Arends AJ, Emmelot-Vonk MH, Melis RJF, Mooijaart SP, et al. [Assessment instruments in frail older patients; a call for more standardisation]. Nederlands tijdschrift voor geneeskunde. 2019;163. 31769625

